# MicroRNA 27b-3p Modulates SYK in Pediatric Asthma Induced by Dust Mites

**DOI:** 10.3389/fped.2018.00301

**Published:** 2018-10-22

**Authors:** Xiaoyan Dong, Nanbert Zhong, Yudan Fang, Qin Cai, Min Lu, Quan Lu

**Affiliations:** ^1^Department of Pulmonary, Shanghai Children's Hospital, Shanghai, China; ^2^Shanghai Institute of Medical Genetics, Shanghai Children's Hospital, Shanghai, China; ^3^Shanghai Children's Hospital, Shanghai, China; ^4^Department of Human Genetics, Institute for Basic Research in Developmental Disabilities, Staten Island, NY, United States; ^5^Chinese Alliance of Translational Medicine for Maternal and Children's Health, Beijing, China; ^6^Peking University Center of Medical Genetics, Peking University Health Science Centre, Beijing, China

**Keywords:** pediatric asthma, cytokines, microRNA, mRNA, dust mites, pathways

## Abstract

The PI3K-AKT pathway is known to regulate cytokines in dust mite-induced pediatric asthma. However, the underlying molecular steps involved are not clear. In order to clarify further the molecular steps, this study investigated the expression of certain genes and the involvement of miRNAs in the PI3K-AKT pathway, which might affect the resultant cytokine-secretion. *in-vivo* and *in-vitro* ELISA, qRT-PCR and microarrays analyses were used in this study. A down-expression of miRNA-27b-3p in dust mite induced asthma group (group D) was found by microarray analysis. This was confirmed by qRT-PCR that found the miRNA-27b-3p transcripts that regulated the expression of SYK and EGFR were also significantly decreased (*p* < 0.01) in group D. The transcript levels of the SYK and PI3K genes were higher, while those of EGFR were lower in the former group. Meanwhile, we found significant differences in plasma concentrations of some cytokines between the dust mite-induced asthma subjects and the healthy controls. On the other hand, this correlated with the finding that the transcripts of SYK and its downstream PI3K were decreased in HBE transfected with miRNA-27b-3p, but were increased in HBE transfected with the inhibitor *in vitro*. Our results indicate that the differential expression of the miRNAs in dust mite-induced pediatric asthma may regulate their target gene SYK and may have an impact on the PI3K-AKT pathway associated with the production of cytokines. These findings should add new insight into the pathogenesis of pediatric asthma.

## Introduction

Pediatric asthma is a common respiratory disease in children worldwide. In China, the incidence of pediatric asthma has increased by approximately 10% in the last decade, while the overall prevalence of asthma among children younger than 14 was 3.02% in urban areas in 2009–2010 (1). Etiologically, asthma may result from gene-environmental interactions, common environmental factors being air pollution, pollen, fungi, and dust mites (2). Up to 80% of asthma patients are allergic to house dust mites, although the exact rate differs in various regions (3). Clinically, in this study, we have focused on dust mite-induced asthma. Asthma as a chronic inflammatory disease, its pathogenic mechanism is complicated. Because the inflammatory reactions are due to the adaptive and innate immune system with the involvement of mast cells, eosinophils, lymphocytes (T, B cells), macrophages, and dendritic cells (4). Moreover, structural cells, epithelial cells and smooth muscle cells may also be involved in the inflammatory environment (5, 6). More importantly, asthma attacks are generally accompanied by a change in the secretion of IgE and cytokines, for example interleukin IL-4, IL-6, Il-12, gamma-interferon (γ-IFN), and tumor necrosis factor (TNF-α) (7–9).

It is known that cytokine secretion is associated with and affected by various signaling pathways including the P13K-AKT and NF-kappaB pathways (10, 11). The PI3K-AKT pathway regulates fundamental cellular functions, for example transcription, translation, proliferation, growth, and survival. A lot of genes were involved in this pathway. So if the expression of upstream or downstream genes in the P13k-AKT pathway is changed, subsequently the biologic processes associated with the PI3K-AKT pathway may also be changed. The latter change may result in a change in cytokine secretions and play a role in the development of diseases, including pediatric asthma (12). It has been reported that differential expression of several genes, including the *SYK* and *EGFR*, which are upstream genes in the inflammatory pathways (PI3K-AKT, NF-kB), may affect the activity of downstream genes in these pathways to influence allergic inflammation diseases (13, 14). Meanwhile, *SYK* and *EGFR* were taken grant for modulating the progress of asthma by function in PI3K pathway and influence on cell differentiation, T or B cell and so on (15). However, it remains unclear how these genes regulate the inflammation pathway associated with the production of cytokines and the occurrence of asthma attacks.

MicroRNAs (miRNAs or miR) are a group of small non–protein-coding RNAs that are 21–25 nucleotides in length. They act as transcriptional regulators involved in many complex human disorders and in biological processes including cell proliferation and apoptosis (16, 17). For example, it showed that miR-223 was involved in the maturation and function of neutrophil differentiation (18). miR-27b-3p was also indicated that influenced breast cancer's therapy by regulating its target gene (19). Thus, the regulation of miRNAs to their targeting genes appears to play an important role in immune inflammatory responses and also in the development of asthma (20). For this reason, we hypothesize that miRNAs may regulate their targeted mRNA(s) involved in the inflammation pathways associated with asthma, leading to an asthma attack. In the current study, we look into the relationships among an inflammation pathway (for example the PI3K-AKT pathway), miRNA(s) and targeted mRNA, in order to better understand the mechanics of the regulation of the production of cytokines and the pathogenesis of pediatric asthma.

## Methods

### Subjects

A total of 150 pediatric patients with asthma were recruited as the Case Group from our Asthma Clinic of Shanghai Children's Hospital. Written informed consents were obtained from these children and their guardians, according to the guidelines of the Bioethics Committee of the Hospital. These 150 Case Group pediatric patients were divided into three groups: (1) Group D: the dust mite-induced asthma children; (2) Group F: the asthma children with food allergy only; (3) Group DF: the dust-mite induced asthma children with food allergy. Enrollment inclusion criteria were: (1) Asthmatic patients older than 4 years who were diagnosed according to the Global Initiative for Asthma guidelines (GINA) (21); (2) Patients without current or recent infection, neither taking any medication, nor had acute asthma exacerbations or attacks before; (3) Total IgE ≥ 60 kU/ L, dust mite allergen-specific serum IgE ≥ 0.35 kU/L. Exclusion criteria were: (1) Patients younger than 4 years; (2) Patients with current or recent infection, taking any medication (such as ICS), or with any acute asthma exacerbations or attacks (Table 1). For the Control Group N, 50 age- and gender-matched healthy children were recruited in this study.

**Table 1:** Demographics of asthma and control groups.

**Group**	**Gender**		**Age (year)**	**IgE (kU/L)**	**Eos (%)**
	**Male**	**Female**			
D	32	18	*6.50 ± 2.72*	453.29 ± 284.28	4.60 ± 2.80
F	31	19	*6.12 ± 2.91*	204.38 *± 194.95*	4.91 *± 2.07*
DF	35	15	*5.30 ± 2.30*	740.76 *± 232.86*	5.84 *± 3.53*
N	28	22	6.30 ± 3.45	38.16 ± 34.51	3.72 ± 3.40

D, dust mite; F, food; DF, dust mite plus food; N, control group. No significance (χ^2^ = 7.271, p = 0.06 > 0.05) observed in gender and age; *IgE, Total IgE; EOS, the percentage of Eosinophils cell in peripheral blood.

### IgE serology

IgE-mediated immune reactions are the most common cause of antigen-induced pediatric asthma. Total IgE and allergen-specific serum IgE, our study inclusion factors (Table 1), were assessed using the ImmunoCAP System (Thermo Fisher Scientific/Phadia AB, Uppsala, Sweden). The cut-off level was 60 kU/L. In the asthma group, total IgE was greater than 60 kU/L. IgE Serology atopic sensitization was indicated if the child had allergen–specific serum IgE ≥ 0.35 kU/L. We tested allergen-specific serum IgE on dust mite and food allergens of Fx5 (egg white, milk, fish, wheat, peanut, and soy) (22).

### Measurement of inflammatory cytokines

The plasma concentrations of IL-4,-6,-10,-12,TNF-α and γ-IFN in the participants were measured using an ELISA kit (Multiscience Biotech Co Ltd, Shanghai, China) with Multiskan MK3 (Thermo, Vantaa, Finland).

### Array hybridization

Twelve pairs of gender- and age-matched Group D dust mite-induced asthma children and Group N normal control children were included in the initial microarray-based discovery analysis. Five milliliter of EDTA-anticoagulant peripheral blood was collected from each child. Total RNA was isolated by using the TRIzol (Invitrogen, Grand Island, NY, USA) and the miRNeasy mini kits (Qiagen, Chatsworth, CA, USA) according to the manufacturer's instructions. This protocol efficiently recovered all RNA species, including miRNAs. The quality and quantity of RNAs collected from all participants were measured with a nanodrop spectrophotometer ND-1000 (Nanodrop Technologies, Wilmington, DE, USA), and RNA integrity was determined by gel electrophoresis. After RNA was isolated from the samples, the miRCURY™ Hy3™/Hy5™ Power labeling kit (Exiqon, Vedbaek, Denmark) was used according to the manufacturer's guidelines for miRNA labeling. Briefly, 1 μg of each sample was 3'-end-labeled with Hy3^TM^ fluorescent dye using T4 RNA ligase by the following procedure: RNA in 2.0 μl of water was combined with 1.0 μl of CIP buffer and CIP (Exiqon, Vedbaek, Denmark). The mixture was incubated for 30 min at 37°C and was terminated at 95°C for 5 min. Then, 3.0 μl of labeling buffer, 1.5 μl of fluorescent label (Hy3^TM^), 2.0 μl of DMSO and 2.0 μl of labeling enzyme were added to the mixture. The labeling reaction was incubated for 1 h at 16°C and was terminated by incubation at 65°C for 15 min. After termination, the Hy3^TM^-labeled samples were hybridized on the miRCURYTM LNA Array (v.18.0) according to the array manual. The total 25 μl mixture from Hy3^TM^-labeled samples with 25 μl hybridization buffer were denatured at 95°C for 2 min, incubated in ice for 2 min, and then hybridized to the microarray at 56°C for 16–20 h with a 12-Bay Hybridization System (Nimblegen, Madison, WI, USA). After hybridization, the slides were prepared, washed with a wash buffer kit (Exiqon, Vedbaek, Denmark) and dried by centrifugation at 400 rpm for 5 min. The slides were then scanned by the Axon GenePix 4000B microarray scanner (Axon Instruments, Foster City, CA, USA).

### miRNA profiling and differential expression analysis

Signal quantification of the microarray's scanned image was captured with GenePix Pro 6.0 software (Axon, Cadiz, Spain). Replicated miRNAs were averaged, and the miRNAs with intensities ≥30 in all samples were chosen to calculate the normalization factor. Expressed data were normalized using the median normalization. Significant differentially expressed miRNAs were identified through volcano plot filtering. Hierarchical clustering was performed to show distinguishable miRNA expression profiling among samples using MEV software (v4.6, TIGR, Cluj-Napoca, Romania). A threshold (fold change ≥2.0 and *p*-value ≤ 0.05) was used to determine the significance of differences of up- or down-regulated miRNAs. The heatmap diagram provided the result of the two-way hierarchical clustering of miRNAs and samples.

### Validation of miRNAs with qRT-PCR

To validate the microarray results, miRNA expression was quantitated in all 200 samples (150 pediatric patients with asthma and 50 normal controls) using quantitative real-time (qRT) PCR. Exiqon product (product no. 206999, Exiqon, Vedbaek, Denmark) was used to validate miRNA-27b-3p, using miRNA-U6 (23) as an internal control. Briefly, reverse transcription was performed using MMLV Reverse Transcriptase (Epicentre, Madison, WI, USA), RNase inhibitor (Epicentre, Madison, WI, USA), 10X buffer (250 mMTris-HCl, pH 8.3; 200 mMKCl; 40 mM MgCl_2_; 5 mM DTT), 2.5 mM dNTP with RT primers at 16°C for 30 min, followed by 42°C for 40 min and 85°C for 5 min. Twenty Microliters of RNA (final concentration of 50 ng/μl) was used as a template. The cDNA products from reverse transcription reactions were quantified using a real-time (RT) PCR System 7500 (Applied Biosystems, Foster City, CA, USA). Amplicon was quantitated by RT- PCR with end-point SYBR green fluorescence. Each assay was performed in triplicate.

### qRT-PCR analysis of the differential expression of mRNAs in children with asthma

*SYK*(Spleen tyrosine kinase) and *EGFR*(Epidermal growth factor receptor) mRNAs were predicted in miRDB for miRNA-27b-3p.Meanwhile, these genes were closely associated with *PI3K* in PI3K-AKT Signaling Pathway (13, 24). So qRT-PCR was performed to determine the expression of these genes in children with dust mite-induced asthma and control groups. Briefly, reverse transcription was performed using MMLV Reverse Transcriptase (Epicentre, Madison, WI, USA), RNase inhibitor (Epicentre, Madison, WI, USA) and SuperScriptTM III Reverse Transcriptase (Invitrogen, Carlsbad, CA, USA). RT primers were added to the reaction mixture at 37°C for 1 min, then at 50°C for 60 min, and finally at 75°C for 15 min. Twenty microliters of RNA (final concentration of 75 ng/μl) was used as template. The cDNA products from reverse transcription reactions were quantified using the Applied Biosystems 7500. Amplicon was quantitated by RT detection of end-point SYBR green fluorescence. Each assay was performed in triplicate. The primers and reaction conditions are listed in Table S1. The relevant quantification was obtained for each sample with normalization to the internal control β-actin.

### Bioinformatics analysis

Web-based programs, including mirBase (http://mirbase.org) and miRDB (http://mirdb.org/miRDB) were employed to predict miRNA target(s). The functional enrichment was analyzed with the DAVID program (http://david.abcc.ncifcrf.gov), in which Gene Ontology and KEGG pathways (http://www.kegg.jp/) were provided for analysis.

### Analysis of the expression of miRNA and the target gene mRNAs in human airway epithelial cells transfected with miRNA plasmid by qRT-PCR and western-blot

To validate the relationship between miRNA and the expression of the target genes *in vitro*, the over-expression miRNA-27b-3p plasmid was constructed and then transfected into human airway epithelial cells 16-HBE (Meilian Biology Company, Shanghai, China). It is known that the 16-HBE cells are the first ones encountered by inhaled dust-mites and involved in the airway inflammation of asthma (5, 25). The inflammation may be mediated by miRNA (26). The human miR-27b-3p gene was amplified with a set of primers (Hsa-miR-27b-3p-R: 5′-CAGGAGACAGTGCATCCTTG-3′ and Hsa-miR-27b-3p-F: 5′-GAACAGGTGCATCTCGTAGC-3′). Then miRNA-27b-3p DNA was conjunct into plasmid pcDNA3.1 (Invitrogen, Carlsbad, CA, USA). The inhibitor of miRNA-27b-3p (cat#4464084, MIRNA INHIB 2.0, 5N STD) was purchased from Ambion, Applied Biosystems or Life Technologies.

HBE was cultured in RPMI Medium 1640 basic (1x) (Gibco, NY, USA) with 10% FBS (Australia-sourced GE Healthcare, Australia), 50 IU/ml penicillin and 50 mg/ml streptomycin (Invitrogen, Paisley, UK). Cells were grown in 6-well plates (1 × 10^5^ cells/well) until they reached 80% confluence.

For transfection, HBE cells were plated at a density of 3.62 × 10^5^ cells/well in 6-well plates. Two microliters of miRNA-27b-3p plasmid or 2 μl of miRNA-27b-3p inhibitor were respectively transfected into the HBEcells with lipofectamine 2000 (Invitrogen, Carlsbad, CA, USA). The un-transfected cells were taken as the blanket control. Each sample was prepared in duplicate, and the entire experiment was repeated three times.

Forty-Eight hours after transfection, the total RNA of cells was isolated using the TRIzol reagent (Invitrogen, Grand Island, NY, USA). Quantitative PCR analysis was performed after mRNA extraction with the above described protocol. Reverse transcription was performed using MMLV Reverse Transcriptase (Epicentre, Madison, WI, USA) and RNase inhibitor (Epicentre, Madison, WI, USA) using SuperScriptTM III Reverse Transcriptase (Invitrogen, Carlsbad, CA, USA). RT primers were added into the reaction mixture at 70°C for 10 min, followed by 37°C for 60 min. Twenty Microliters of RNA (final concentration of 75 ng/μl) was used as template. The cDNA products from reverse transcription reactions were quantified using the Applied Biosystems 7500. Amplicon was quantitated by RT detection of end-point SYBR green fluorescence. Each assay was performed in triplicate. Before mRNA PCR analysis, we also tested the level of miRNA-27b-3p expression in all samples of HBE cells by RT-PCR to assess the transfection efficiency in HBE.

Forty-Eight hours after transfection, Protein samples were also collected from 16HBE transfected with plasmid pcDNA3.1,normal control cell (NC) and HBE with miRNA-27b-3p inhibitor, KB. The concentration of every protein samples would be measured by BCA assay (Beyotime, Shanghai, China) after cell lysis by RIPA lysis buffer (Beyotime, Shanghai, China). According to the concentration, we put 40 μg protein sample in per gel well which we have prepared first and added molecular marker per gel well. Then running the gels at 100 V for 20 min, then change to 120 V Constant voltage electrophoresis until the marker at the end of gel. After that time, we transferred membranes (iBlot Dry Blotting System, Thermo Scientific, Rehovot, Israel) based on the instruction of product. The membrane was placed in Tris buffered saline tween (TBST) solution containing 5% skim milk powder and incubated at room temperature for 1 h. According to protocol of antibody SYK, anti-EGFR, anti-GAPDH (Packaging Mix, Shanghai, China), put primary antibody and secondary antibody one after another, then exposing membrane by X ray (film from FF507, KODAK, and New York, USA).

### Statistical analysis

The differences between the groups were analyzed by Student's *t* test when two groups were compared. It will be used by one-way ANOVA when more than two groups were compared. Analyses were performed with SPSS 19.0 (IBM, Armonk, NY, USA). *p* < 0.05 was considered statistically significant.

## Results

### The demographics of the asthma group and control, and increased IgE in the asthma group

Our Case Group D had 32 boys and 18 girls with the average age of 6.50 ± 2.72 years old. During the time they were enrolled in this study, their blood specimens were collected. Group F had 31 boys and 19 girls with the average age of 6.12 ± 2.91 years old. Group DF had 35 boys and 15 girls with the average age of 5.30 ± 2.30 years old. Our Normal Group N had 28 boys and 22 girls with the average age of 6.30 ± 3.45 years old. No significant difference was observed between the Case Groups and the Control Group in terms of gender and age (χ^2^ = 7.271, *p* = 0.06 > 0.05) (Table 1).

**Figure 1:**
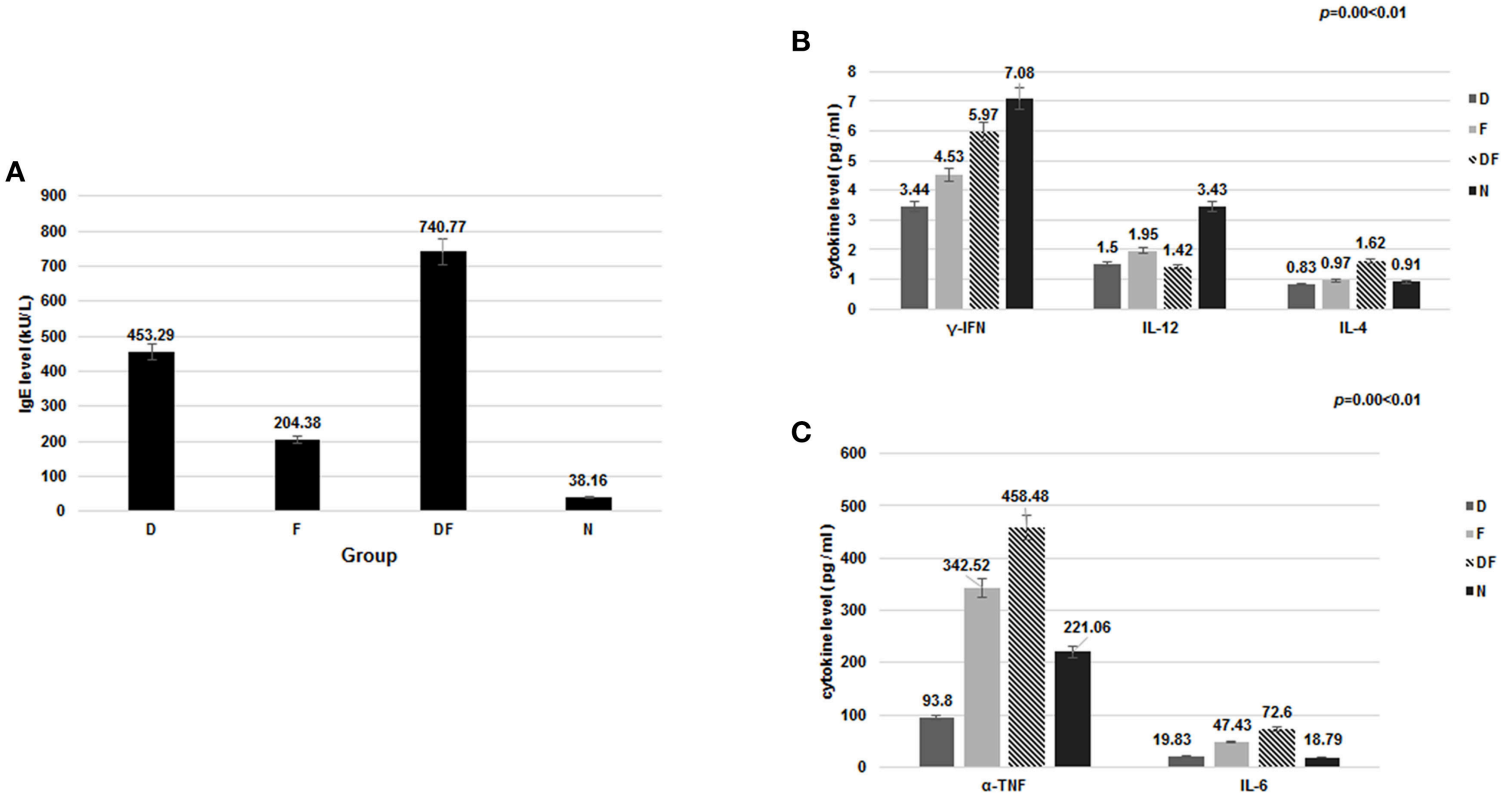
The level of IgE and cytokine in the four groups [dust mite (D), food (F), dust mite plus food (DF), normal control group (N)]. **(A)** Total IgE was increased in the dust mite, food and dust mite with food groups compared with normal controls. **(B)** significant decrease in plasma γ-IFN and IL-12 was found in Group D, Group F and Group DF, compared with Group N (*p* < 0.01). IL-4, IL-6, and TNF-α were increased in Group DF (*p* < 0.01). **(C)** TNF-α and IL-6 were also increased in the Group F, except in Group D (*p* < 0.05).

We found that total IgE was increased in all asthma Groups when compared with non-asthma Normal Group. Within the asthma Groups, the combination group of dust mite with food Group DF showed the highest level of IgE, followed by the dust mite alone Group D and food alone Group F (Table 1, Figure 1A).

### ELISA analysis of the concentrations of related inflammatory cytokine

It is known that inflammatory cytokine factors γ-IFN, IL-12, IL-4, IL-6, and TNF-α are related to airway inflammation and the PI3K-AKT pathway (27, 28). In the current study, significant decrease in plasma γ-IFN was found in Group D (3.44 ± 2.87 pg/ml), Group F (4.53 ± 3.06 pg/ml) and Group DF (5.97 ± 4.17 pg/ml), compared with Group N (7.08 ± 4.30 pg/ml) (*p* < 0.01). IL-12 showed a similar decrease in Group D (1.50 ± 0.66 pg/ml), Group F (1.95 ± 1.56 pg/ml) and Group DF (1.42 ± 1.56 pg/ml) vs. Group N (3.43 ± 1.37 pg/ml) (*p* < 0.01). IL-4, IL-6, and TNF-α were increased, however, in Group DF (IL-4: 1.62 ± 1.13 pg/ml vs. 0.91 ± 0.78 pg/ml; IL-6: 72.60 ± 65.52 pg/ml vs. 18.79 ± 12.64 pg/ml; TNF-α: 458.48 ± 406.42 pg/ml vs. 221.06 ± 150.33 pg/ml, respectively, *p* < 0.01). TNF-α and IL-6 were also increased in the Group F (342.52 ± 310.88 pg/ml vs. 221.06 ± 150.33 pg/ml; 47.43 ± 40.24 pg/ml vs. 18.79 ± 12.64 pg/ml, respectively, *p* < 0.05) (Figures 1B,C).

### Differential expression of miRNA-27b-3p in dust mite-induced asthma

**Figure 2:**
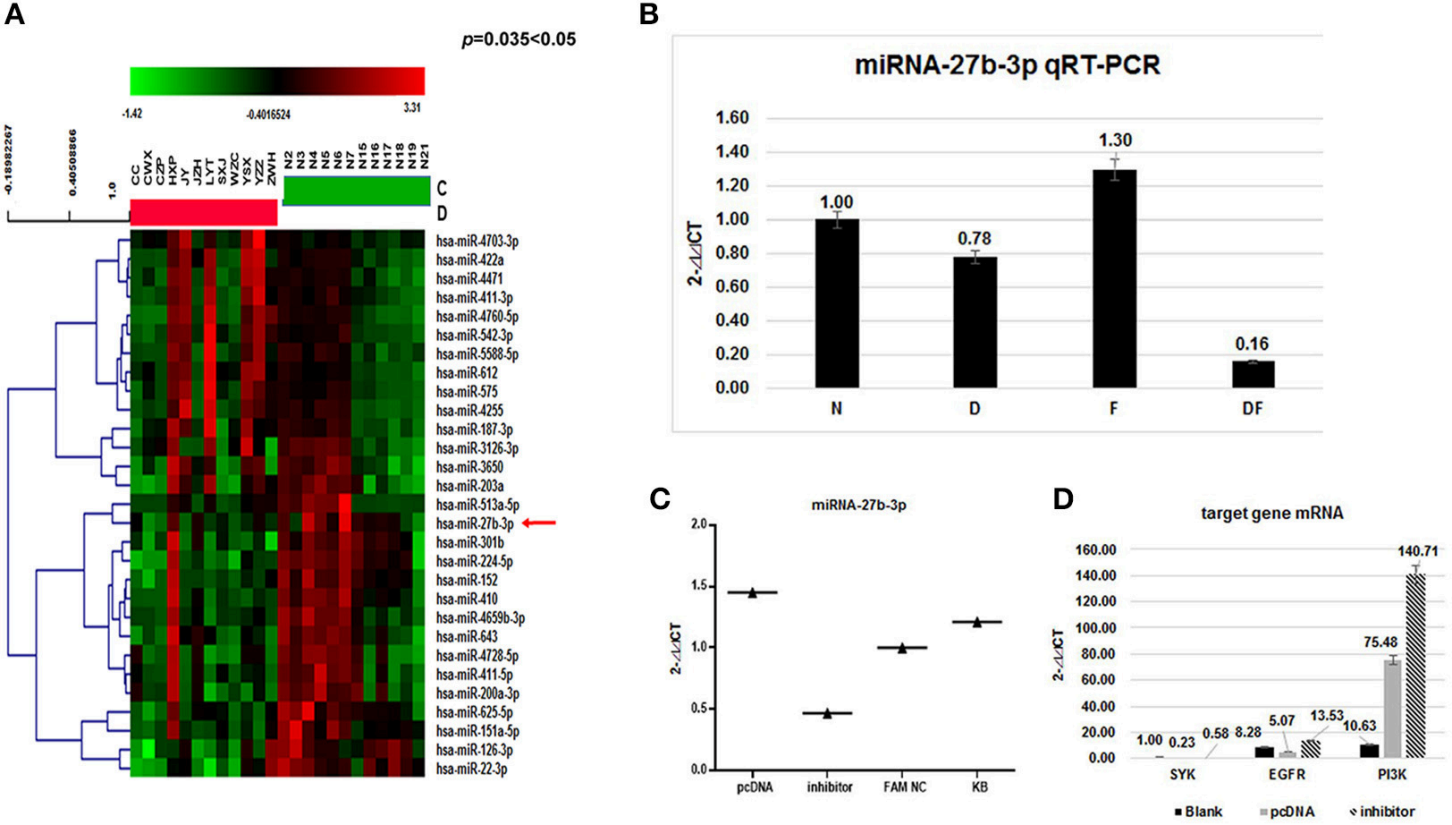
Validation of miRNAs and target gene mRNA in the four groups and HBE. **(A)** The heat map shows a main part of the clustering of miRNAs. Red indicates high relative expression, and green indicates low relative expression. miR-27b-3p was 2.5-fold down-regulated in the asthma group compared with the control group (*p* < 0.05); **(B)** To validate the microarray results, miRNA expression was quantitated in all 200 samples using quantitative real-time (qRT) PCR. The results showed that the average levels of miR-27b-3p in the dust mite group and the dust mite plus food asthma group were significantly lower than those in the control group (*p* = 0.00 < 0.01). Specifically, the expression of miRNA-27b-3p in the dust mite plus food group was the lowest (*p* = 0.00 < 0.01); **(C)** miRNA-27b-3p expression after transfection with miRNA 27b-3p plasmid (pcDNA) and its inhibitor in HBE cells. The KB group contained blank HBE as the control. The expression of miRNA-27b-3p was increased in HBE transfected with miRNA 27b-3p plasmid and decreased in HBE with the inhibitor. **(D)** The expressions of *SYK, EGFR*, and *PI3K* were increased more in transfected with miRNA-27b-3p inhibitor than with miRNA 27b-3p plasmid in HBE (*p* < 0.05).

Among differentially expressed miRNAs, 112 were identified as up-regulated and 10 were down-regulated (Figure 2A) (29). The expression of miRNA-27b-3p, functionally linked to inflammation and apoptosis by bioinformatics analysis, showed a 2.5-fold decrease and had statistical significance (*p* = 0.035 < 0.05). This miRNA-27b-3p was applied to predict targeting transcripts in mirBase (http://mirbase.org) and miRDB (http://mirdb.org/miRDB). SYK and EGFR mRNAs were predicted in miRDB for miRNA-27b-3p. This suggests that miRNA-27b-3p may have a regulatory function involved in the degradation or inhibition of SYK and EGFR in the asthma group(s).

**Figure 3:**
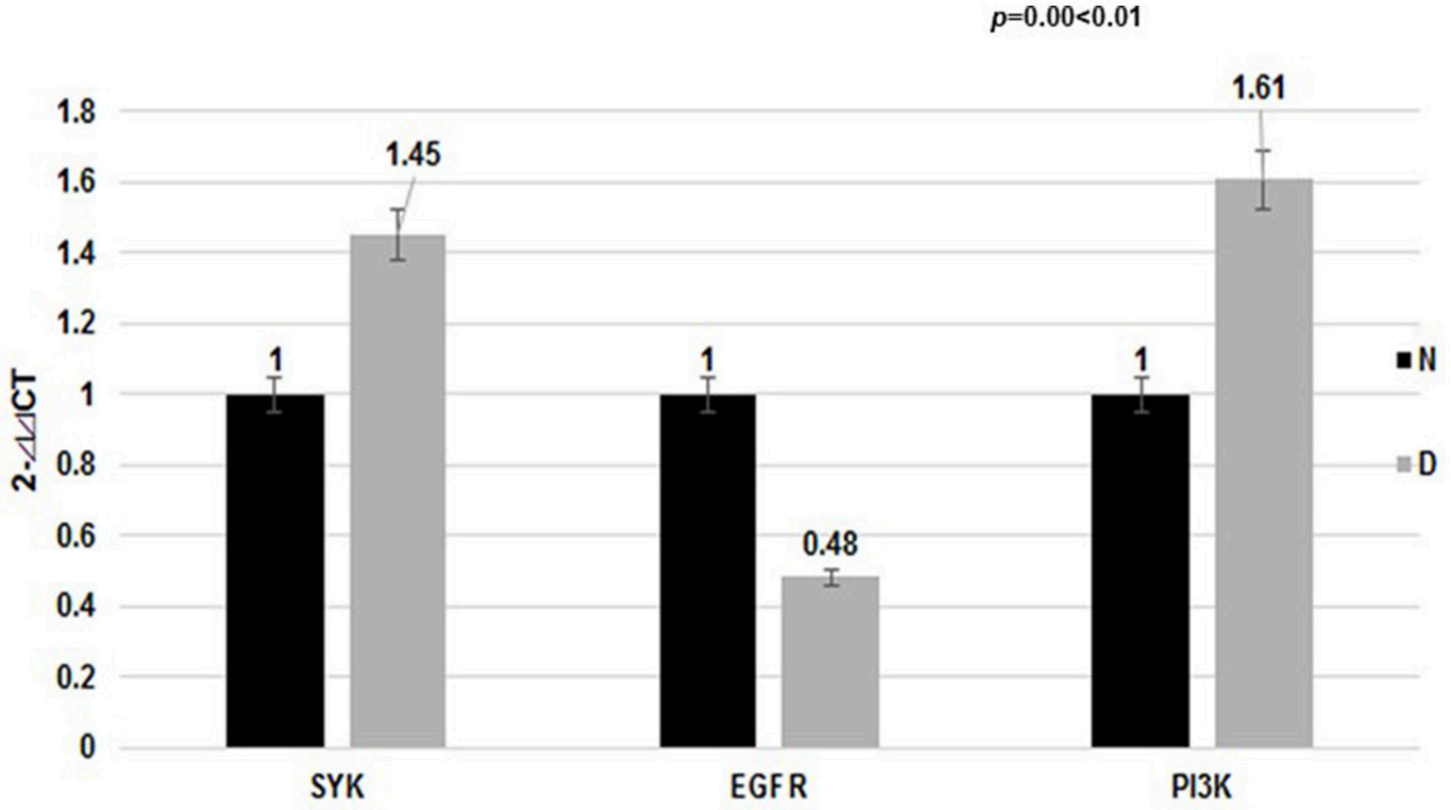
Expression of target mRNA in the dust mite group and normal control group. The EGFR mRNA level in the dust mite-induced asthma group was lower compared with the control group (*p* < 0.01). The SYK mRNA level was elevated (*p* < 0.01). The mRNA level of their downstream gene (PI3K) was differentially elevated in the dust mite-induced asthma group compared with the control group (*p* = 0.00 < 0.01).

To further confirm the miR-27b-3p data from the microarray analysis, the same independent 150 asthma children (50 cases in each group of D, F, and DF) and 50 normal controls (N) were subjected to qRT-PCR to validate the data generated from the microarray. Our results showed that the average levels of miR-27b-3p in the dust-mite (Group D) and dust-mite with food allergy (Group DF) were significantly lower than that in the control Group N, except the level in food allergy group (Group F) was little higher (D group: 0.78 vs. 1; DF group: 0.16 vs. 1, F group: 1.3 vs. 1, *p* = 0.00 < 0.01) (Figure 2B). There was also statistical significance observed among Group D, Group DF, and Group F (*p* < 0.05) by ANOVA analysis. Specifically, the expression of miRNA-27b-3p in Group DF was the lowest (*p* = 0.00 < 0.01) (Figure 3B). This suggests that miRNA-27b-3p maybe more regulate in dust mite-induced asthma.

### qRT-PCR analysis of the expression of *EGFR* and *SYK* mRNAs in dust mite-induced asthma

Meanwhile, our data showed that the *EGFR* mRNA level in Group D was lower than that in the control Group N (2-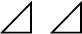
ct: 0.48 vs. 1.00, *p* < 0.01), while the *SYK* mRNA level was higher (2-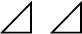
ct: 1.45 vs. 1.00, *p* < 0.01). We found that the mRNA level of the downstream gene (PI3K) was differentially elevated in the dust mite-induced asthma Group D (Figure 3) compared with the normal control Group N (2-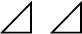
ct: 1.61 vs. 1.00, *p* = 0.00 < 0.01).

### Validation of the regulation of miRNA-27b-3p to the target gene in HBE

To validate the relationship of miRNA-27b-3p and its target mRNA gene (*SYK, EGFR*) *in vitro*, we further measured the transcript levels of the genes (*SYK, EGFR*, and *PI3K*) in HBE cells transfected with miRNA-27b-3p plasmid or its inhibitor by qRT-PCR. We found that the transcripts of *SYK, EGFR* and *PI3K* in miRNA-27b-3p transfected cells were significantly decreased but they were increased in transfected cells with inhibitor (*SYK*: 0.11 vs 0.55; *EGFR*: 0.29 vs. 0.68; *PI3K*: 3.22 vs. 12.45,*p* < 0.05) (Figures 2C,D). The above results indicated that miRNA-27b-3p might downgrade the expression of *SYK, EGFR* and their downstream gene *PI3K* in the PI3K-AKT pathway. Once the suppression of miRNA 27b-3p expression resulted in the expression of the target genes of *SYK, EGFR* and the downstream genes, *PI3K* was increased. These results are consistent with our observations in patients with dust mite-induced asthma.

**Figure 4:**
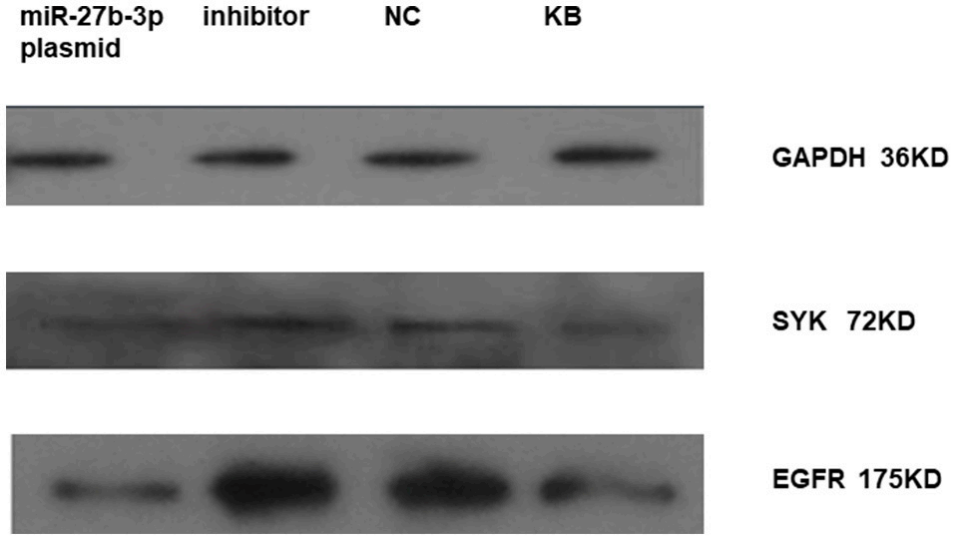
Expression of *SYK* and *EGFR* in HBE transfected with miRNA 27b-3p plasmid (pcDNA) by western-blot. It showed that the expression of *SYK* and *EGFR* in HBE transfected with miRNA-27b-3p plasmid were lower compared to HBE transfected with miRNA-27b-3p inhibitor and normal control (NC) in the picture. The blot of *EGFR* was clearly deeper in HBE transfected with inhibitor and NC compared to *SYK*.

We found that miRNA-27b-3p influenced on the expression of *SYK* and *EGFR* in HBE by western-blot as well. It showed that the expression of *SYK* and *EGFR* in HBE transfected with miRNA-27b-3p plasmid was lower compared to HBE transfected with miRNA-27b-3p inhibitor and normal control (NC) (Figure 4). The blot of EGFR was clearly higher than the other in HBE transfected with miRNA-27b-3p inhibitor.

## Discussion

**Figure 5:**
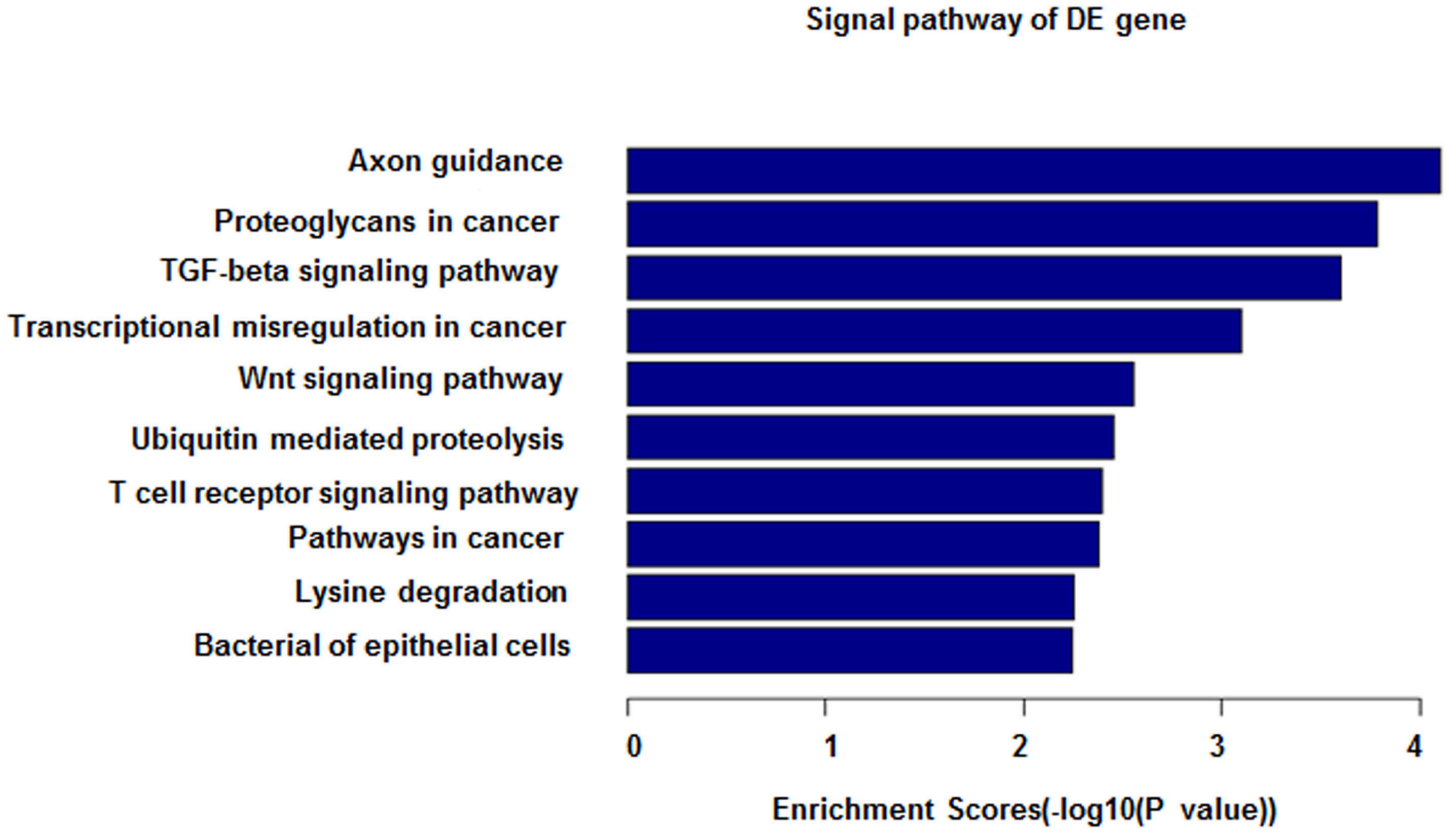
miRNA-27b-3p function analysis in GO analysis. The horizontal axis shows the enrichment scores in the GO analysis. From this figure, it can be seen that miRNA-27b-3p was associated with the TGF-beta signaling pathway, Wnt signaling pathway and T-cell signaling pathway; all of their enrichment scores were above 2.0.

In our study, we have found that miRNA-27b-3p is down-expressed in pediatric asthma, especially in dust-mite allergen-induced pediatric asthma. As an immune regulator, miRNA appears to play an important role in the regulation of immune reactions and the inflammatory signaling pathway in pediatric asthma (30). Previous studies have shown that miRNA can control the inflammation pathway leading to asthma attacks by mediating target gene expression (31). The function of miRNA represents the regulation of decreased expression of their target gene mRNAs as well as mRNA degradation. From our study, miRNA-27b-3p is also related to the inflammation pathway by bioinformatics analysis (Figure 5). By miRDB analysis, *SYK* and *EGFR* are target genes of miRNA-27b-3p. Meanwhile, *PI3K* is a downstream gene of *SYK* and *EGFR* in the PI3K-AKT pathway. Thus, we think that miRNA-27b-3p is important in the PI3K-AKT pathway, although this has not been reported in previous studies. In our study, we have also found the decreased expression of miRNA-27b-3p and increased expression of *SYK* and *PI3K*. As a target gene of miRNA-27b-3p by miRDB analysis, the expressions of *SYK* and *PI3K* (a downstream gene of *SYK* and *EGFR* in the PI3K-AKT pathway) were significantly increased in the asthma group compared with the control group (*p* < 0.05). At the same time, the levels of several cytokines changed. The levels of γ-INF and IL-12 were decreased in the dust mite and dust mite with food allergy groups. The levels of IL-4 and IL-6 were significantly increased in dust mite plus food group. We know that γ-INF and IL-12 are mediated by Th1, while IL-4 and IL-6 mediated by Th2. These different levels of production of cytokines are related to the Th1/Th2 imbalance that induces asthma attacks (32). Thus, it appears that the expression of these gene (*SYK, EGFR, PI3K*), which are involved in the PI3K-AKT pathway, may be connected with the secretion level of cytokine in asthma, especially in dust-mite induced pediatric asthma.

From above, it showed that the significantly difference of expression of miRNA and mRNA were gotten in dust mite induced asthma group. Dust mite-induced pediatric asthma, as is well-known, is a clinical phenotype of asthma which is the most common allergen induced pediatric asthma and adult asthma (33). Frequent exposures to house dust mite (HDM) allergen concentrations during early life play a role in the development of allergic sensitization (3). The latter induces multifaceted immune responses in the adaptive and innate immune systems (25). During these responses, a number of pathways (PI3K-AKT, JAK-STAT, NF-κB, and MAPK) are associated with T- and B-cell differentiation and cytokine secretion (34–36). The PI3K-AKT pathway is the most important among these pathways, possibly influencing the JAK-STAT, MAPK and the downstream NF-κB (37–39). Previous studies have shown that *SYK* and *EGFR* are related to the inflammation pathway PI3K-AKT, which influences the differentiation and maturity of B and T cells (13, 40, 41). *SYK* (Spleen tyrosine kinase) plays a key role in the inflammation pathway of B cells and Fc receptors (42) and affects mast cells and cytokine secretion (40, 43). Because *PI3K* is a downstream gene of *SYK, SYK* can induce the activity of *PI3K*. Therefore, it is most likely that the PI3K-AKT pathway is also involved in these inflammation reactions, as indicated by some studies (14, 24). Meanwhile, it also has been shown that *EGFR* (Epidermal growth factor receptor) can regulate the inflammation pathway in pulmonology allergy disease and asthma (13). In our study, we have found that there are significant differences in the expression of genes (*SYK, PI3K, and EGFR*) and the concentration of cytokines between dust mite-induced asthma and healthy controls, suggesting that *EGFR, SYK*, and *PI3K* may be involved in dust mite-induced pediatric asthma. However, this result does not clearly explain the process of the regulation of these genes to produce cytokines.

In our study, we found that the decreased expression of miRNA-27b-3p in dust mite-induced pediatric asthma as well as the increased expression of *SYK*. We speculated the decreased expression of miRNA-27b-3p may reduce the degradation of the target gene *SYK*, which then alters the activity of *PI3K* and affects the production of cytokines, finally leading to an asthma attack. Results of HBE cells transfected with the miRNA-27b-3p plasmid in this study showed that the increased expression of miRNA-27b-3p resulted in decreased expression of target genes *SYK* and *EGFR*, even *PI3K* by qRT- PCR and western-blot,which adds to the support of the relationship between miRNA and target gene.

miRNA-27b-3p belongs to the miRNA-27 family located on 9q^22.32^. Several studies about its function in liver enzyme, lipid metabolism, the cardiovascular system and cancer have been published (44–46). At present, there were researches about increased expression of miRNA 27b-3p in animal model of asthma (47). In this study, we found that the expressions of miRNA-27b-3p are decreased (*p* ≤ 0.01) in children with asthma. miRNA-27b-3p expression is decreased only in the dust-mite and dust-mite plus food groups but not in the food group. This suggests that the discrepancy of the expressions of miRNA-27b-3p may have an important function in dust mite-induced pediatric asthma. On the other hand, it is interesting that the expression of miRNA-27b-3p in the dust mite with food allergy group is the lowest (*p* = 0.00 < 0.01). Meanwhile, the levels of IL-4 and IL-6 are significantly increased in the dust mite plus food group and are not changed in dust mite group. These changes may explain why children with dust mite plus food allergies have more allergic symptoms, for example, eczema, and allergic rhinitis (48), along with asthma or severe asthma. Thus, we hypothesize that the dust mite plus food allergy may further influence the expression of miRNA-27b-3p via a superimposed effect. We plan to follow up with studies to determine if food protein can link to dust mite protein to form a new structure to affect the expression of miRNA-27b-3p.

In contrast, the expression of *EGFR* is decreased both in dust mite-induced asthma patients and HBE transfected with miRNA-27b-3p plasmid, which is not we have expected. According to the rule of miRNA regulating targeted mRNA, in which targeted mRNA can be regulated by multiple miRNAs, we propose that *EGFR* may be co-regulated by other miRNAs in dust mite-induced asthma. Of course, this speculation needs to be studied further in the future.

**Figure 6:**
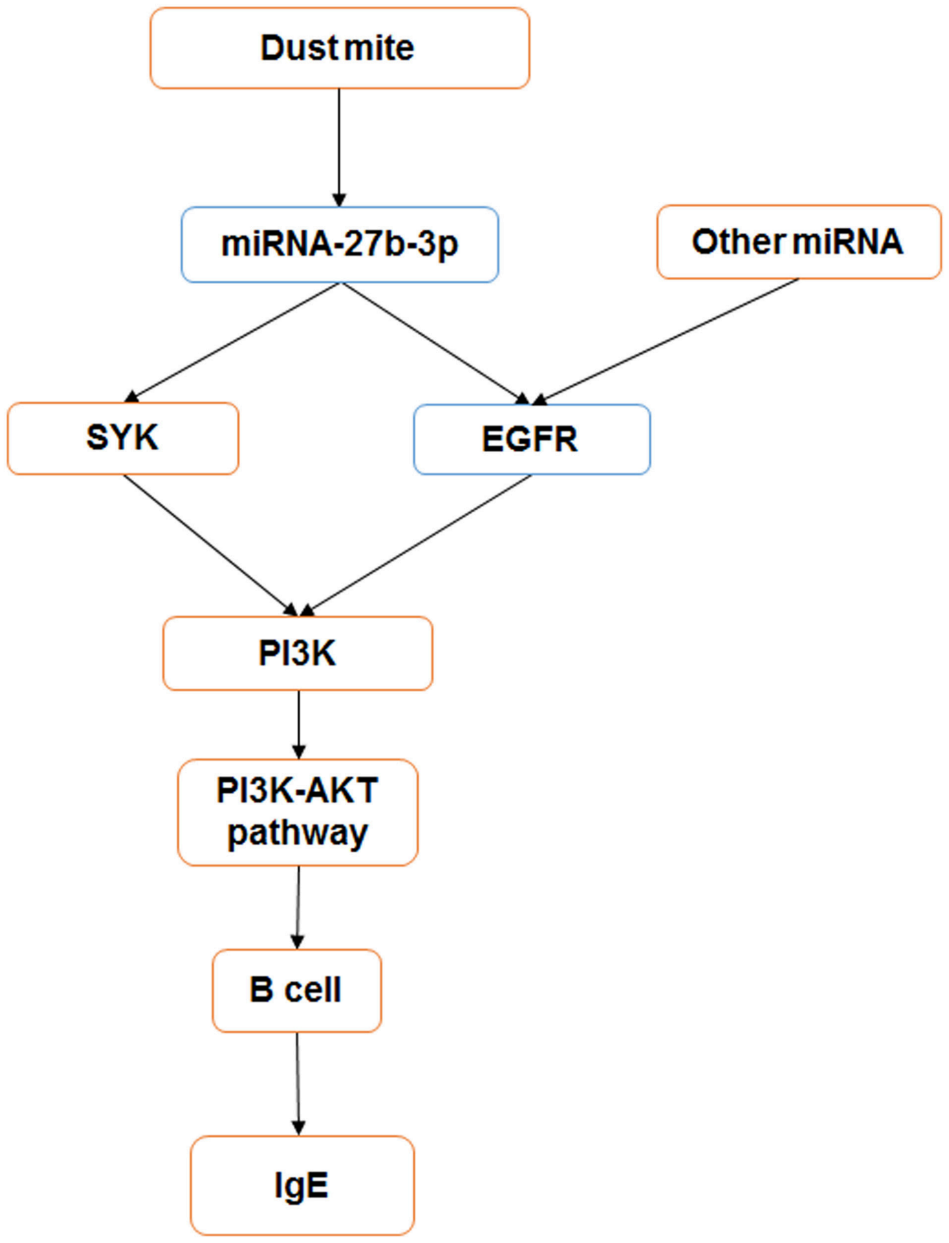
miRNA-27b-3p regulated target genes and the PI3K- Akt pathway in dust mite-induced pediatric asthma. miRNA-27b-3p could regulate its target genes *SYK* and *EGFR*, which induced the expression of the downstream gene PI3K. Then, the activity of the PI3K-akt pathway was changed, and the process of B-cell secreting IgE was influenced in turn. Blue indicates the expression is decreased, and orange indicates it is increased.

In summary, our study shows that miRNA-27b-3p may play an important role in the mediation of the immune reaction in dust mite-induced pediatric asthma. It may regulate the target genes *SYK* and *EGFR* to influence the PI3K-AKT pathway and alter cytokine secretion (Figure 6).

## Author contributions

XD participated in the design of the study, carried out qPCR, western-blottechnique and interpreted the data and wrote the manuscript. YF carried out primer designed and qPCR technique. ML and QL contributed samples and clinical data. QC carried out cell culture. NZ participated in the design of the study, interpreted the data and reviewed the manuscript. All authors read and approved the final manuscript.

### Conflict of interest statement

The authors declare that the research was conducted in the absence of any commercial or financial relationships that could be construed as a potential conflict of interest.
