# Supplementary material for: MicroRNA 27b-3p Modulates SYK in Pediatric Asthma Induced by Dust Mites
Source: Front Pediatr. 2018 Oct 22;6:301. doi: 10.3389/fped.2018.00301 (PMC6204538; doi:10.3389/fped.2018.00301)
Supplement: Supplementary file 1 [file Table_1.DOCX]

**Table S1：Primers used in qRT-PCR of mRNA**

| **Name of gene** | **Bidirectional**  **primer sequence** | **Annealing temperature (^o^C)** | **Amplicon length (bp)** |
| --- | --- | --- | --- |
| β-actin | F:5’CCTGTACGCCAACACAGTGC3'  R:5’ATACTCCTGCTTGCTGATCC3’ | 60 | 211 |
| SYK | F:5’GTGCGGATGATCGGGATAT3'  R:5’TCTTGGCGTAATGTTGGGTAA3' | 60 | 232 |
| PI3K | F:5’TGCCTGCGACAGATGAGTGA3'  R:5’GCCCTATCCTCCGATTACCA3' | 60 | 139 |
| EGFR | F:5’CCAAGGCACGAGTAACAAGC3'  R:5’ATTCCCAAGGACCACCTCAC3' | 60 | 100 |
